# *Cymbalcloeon* gen. nov., an incredible new mayfly genus (Ephemeroptera: Baetidae) from Thailand

**DOI:** 10.1371/journal.pone.0240635

**Published:** 2020-10-13

**Authors:** Chanaporn Suttinun, Jean-Luc Gattolliat, Boonsatien Boonsoong

**Affiliations:** 1 Animal Systematics and Ecology Speciality Research Unit (ASESRU), Department of Zoology, Faculty of Science, Kasetsart University, Bangkok, Thailand; 2 Museum of Zoology, Lausanne, Switzerland; 3 Department of Ecology and Evolution, Lausanne University, Lausanne, Switzerland; Museum National d'Histoire Naturelle, FRANCE

## Abstract

The genus *Cymbalcloeon* gen. nov. (Ephemeroptera: Baetidae) is established for a new species *Cymbalcloeon sartorii* sp. nov. from Thailand, based on larval stage. This genus is unique among all of the Baetidae by the presence of three pairs of single gills on segments V–VII, ventrally oriented to cover the sterna VI–IX. *Cymbalcloeon sartorii* gen. nov. *et* sp. nov. presents unique or rare morphological characters such as a deeply concave margin between the prostheca and mola, without setae; a very large subtriangular process of the left mandible; a maxillary palp segment II with scarce and very long setae; almost completely fused labial palp segments II and III with numerous very long setae; elongate tarsal claw with two rows of teeth; a shagreen surface of the terga and paraproct; and a very reduced body size. The gills of the new genus move very quickly during respiration and present a near-synchronous protraction. Due to the very derived larval morphological character and the unknown imaginal stage, the exact phylogenetic position of the genus remains unclear; it most certainly belongs to the concept of Anteropatellata and is possibly closely related to the genus *Baetopus*.

## Introduction

The family Baetidae is the most diverse family among mayflies, comprising 1,070 species in 110 genera [1, 2]; this accounts for about 30% of all mayfly species worldwide. The family is distributed worldwide, except for Antarctica and New Zealand. The number of studied species in this family in Southeast Asia has steadily increased in the last decade, with about 45 species and 4 new genera described from this area. Most of these mayflies have been reported from islands. For example, forty-seven new species of *Labiobaetis* Novikova & Kluge, 1987 were report from Indonesia, New Guinea, and Malaysia (Sabah) [3–5]. *Procerobaetis* Kaltenbach & Gattolliat, 2020 is the last described genus of Baetidae, with 3 new species from Southeast Asia, discovered in the Philippines and Indonesia [6]. However, the number of species known from this area most certainly remains much lower than the real diversity [7].

In the present study, we describe an incredible new genus of the family Baetidae from Thailand based on larval stage only. A combination of morphological characters distinguishes this new genus from all other known Baetidae genera; the new genus presents a rather unusual habitus. Its gill morphology and gill movements are highly remarkable.

## Materials and methods

### Morphological observations

Specimens were collected from 2 localities, out of 105 localities included in a survey of Baetidae from Thailand, from May 2017 to July 2020. Baetid larvae were collected by kicking method in a slow-flowing area with D-frame nets from the Mae Klong headwater stream (14°34’57.9”N 098°34’52.0”E) and the Loei headwater stream (17°06’24.0”N 101°28’43.4”E). Specimens were preserved in 95–99% ethanol then separated to generic or specific levels in the laboratory. In addition, some live specimens were sorted, taken in photo, and recorded in video in the field using stereo microscope. Five larvae belonging to the new taxon were dissected and mounted on slides in Euparal mounting medium. Photographs were taken using an Olympus BX43 microscope. Final plates were generated using Adobe Photoshop 2020. The material examined is housed in the Aquatic Insects Collection (AIC) of the Zoological Museum Kasetsart University (ZMKU), Bangkok, Thailand, and in the Museum of Zoology, Lausanne, Switzerland (MZL).

Drawings were made using an Olympus BX43 microscope. Photographs of larvae were taken using NIKON SMZ445 and Canon EOS 6D camera and the Visionary Digital Passport imaging system (http://www.duninc.com) and processed with Adobe Photoshop Lightroom (http://www.adobe.com) and Helicon Focus version 5.3 (http://www.heliconsoft.com). Video was recorded using iPhone 11 Pro Max via a NIKON SMZ445 stereo microscope.

### Ethics statement

We followed all guidelines of the Animal Care & Use Guidelines of Institutional Animal Care and Use Committee of Kasetsart University (IACUCKU, approval no. ACKU63-SCI-006) for collecting the mayfly specimens. Study areas are public area which no specific permissions were required for these locations. This work did not affect to endangered or protected species. And all these must be respected and cared for habitat destruction.

### Nomenclatural acts

The electronic edition of this article conforms to the requirements of the amended International Code of Zoological Nomenclature, and hence the new names contained herein are available under that Code from the electronic edition of this article. This published work and the nomenclatural acts it contains have been registered in ZooBank, the online registration system for the ICZN. The ZooBank LSIDs (Life Science Identifiers) can be resolved and the associated information viewed through any standard web browser by appending the LSID to the prefix "http://zoobank.org/". The LSID for this publication is: urn:lsid:zoobank.org:pub:5EF3EF02-9006-4F6C-A2F5-89586FDF94EB. The electronic edition of this work was published in a journal with an ISSN; it has been archived and is available from the following digital repositories: PubMed Central, LOCKSS.

## Results

### *Cymbalcloeon* gen. nov.

*urn:lsid:zoobank.org*:*act:C3B9CB7D-F558-482D-BEDE-AB940E17524B*

#### Type species

**Fig 1:**
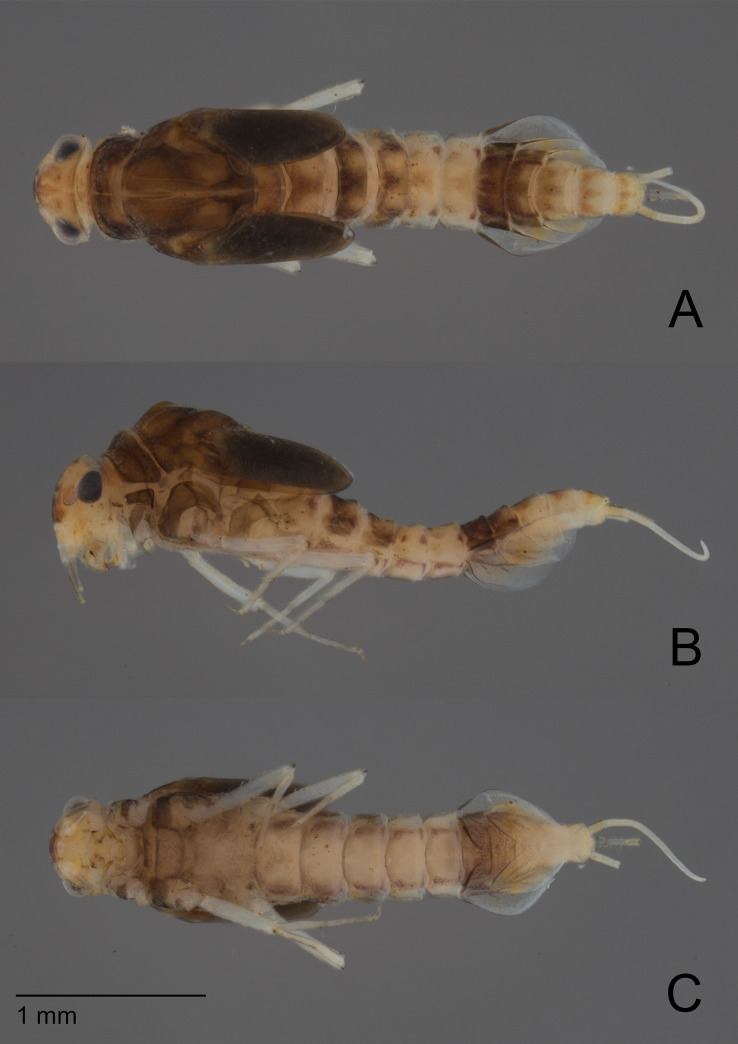
*Cymbalcloeon sartorii* gen. nov. *et* sp. nov. Female mature larva. (A) dorsal view, (B) lateral view, (C) ventral view.

**Fig 2:**
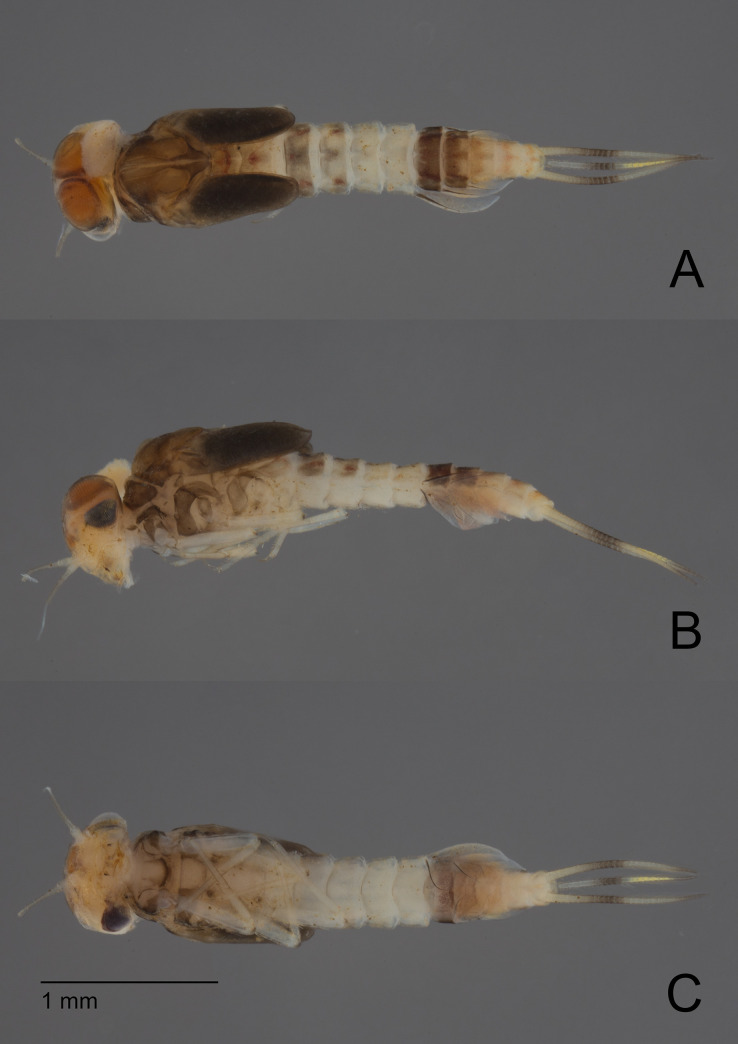
*Cymbalcloeon sartorii* gen. nov. *et* sp. nov. Male mature larva. (A) dorsal view, (B) lateral view, (C) ventral view.

**Fig 3:**
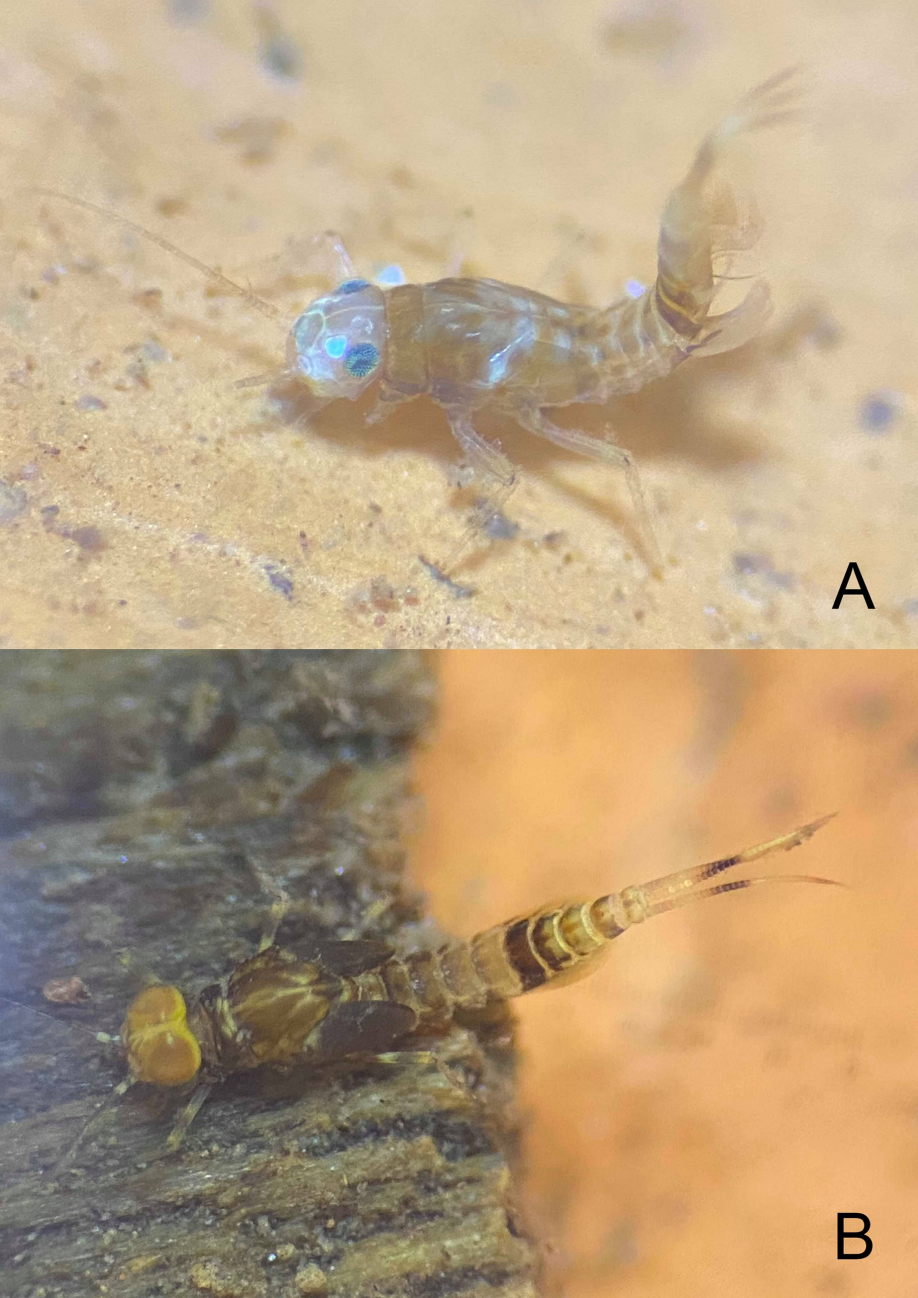
*Cymbalcloeon sartorii* gen. nov. *et* sp. nov. Larva habitus. (A) female larva, (B) male mature larva.

*Cymbalcloeon sartorii* sp. nov. (Figs 1–3), by present designation.

#### Diagnosis

**Fig 4:**
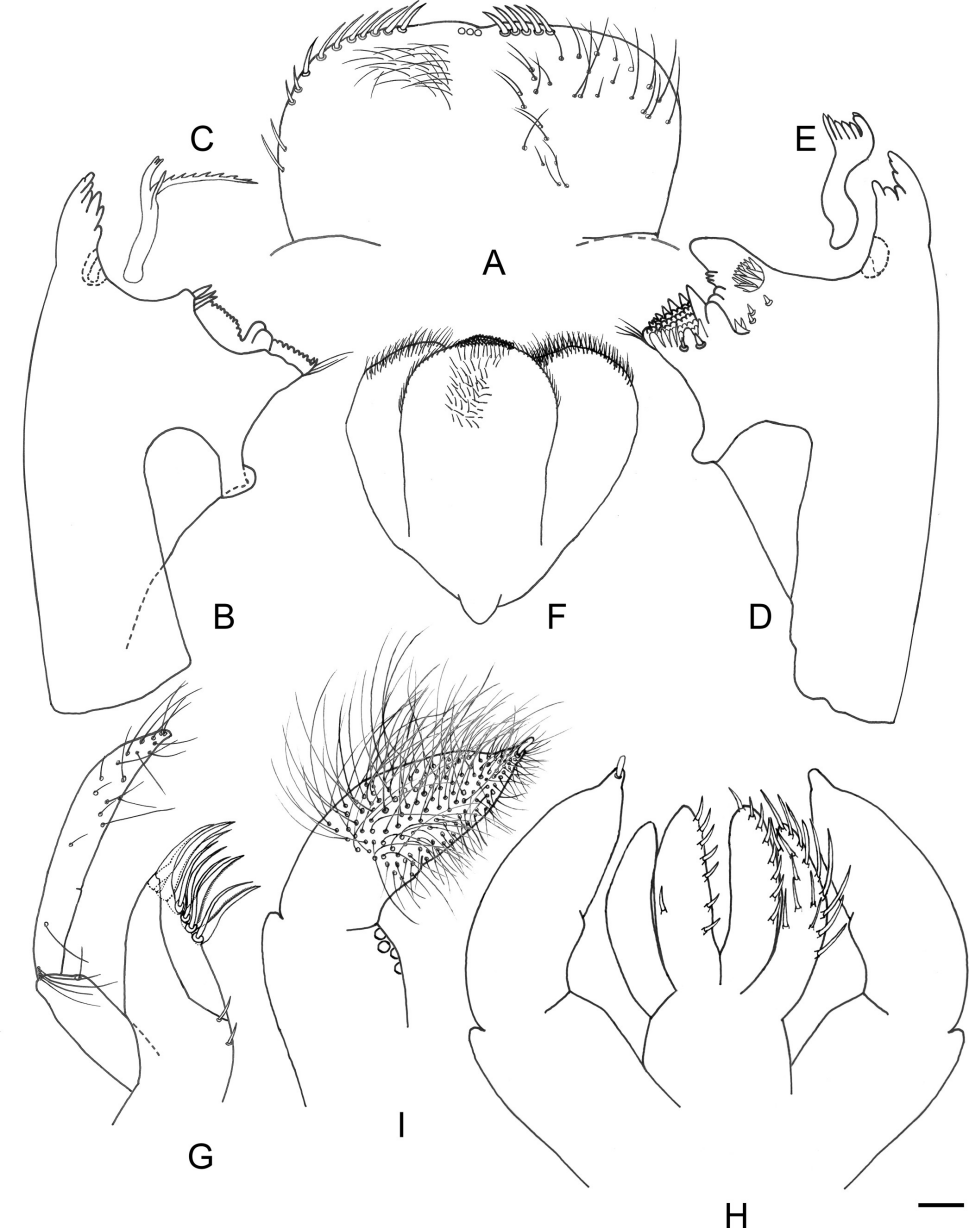
*Cymbalcloeon sartorii* gen. nov. *et* sp. nov. Mouthpart morphology. (A) labrum, (B) right mandible, (C) right prostheca, (D) left mandible, (E) left prostheca, (F) hypopharynx, (G) maxilla, (H) labium, (I) labium palp. Scale bar: 0.02 mm.

**Fig 5:**
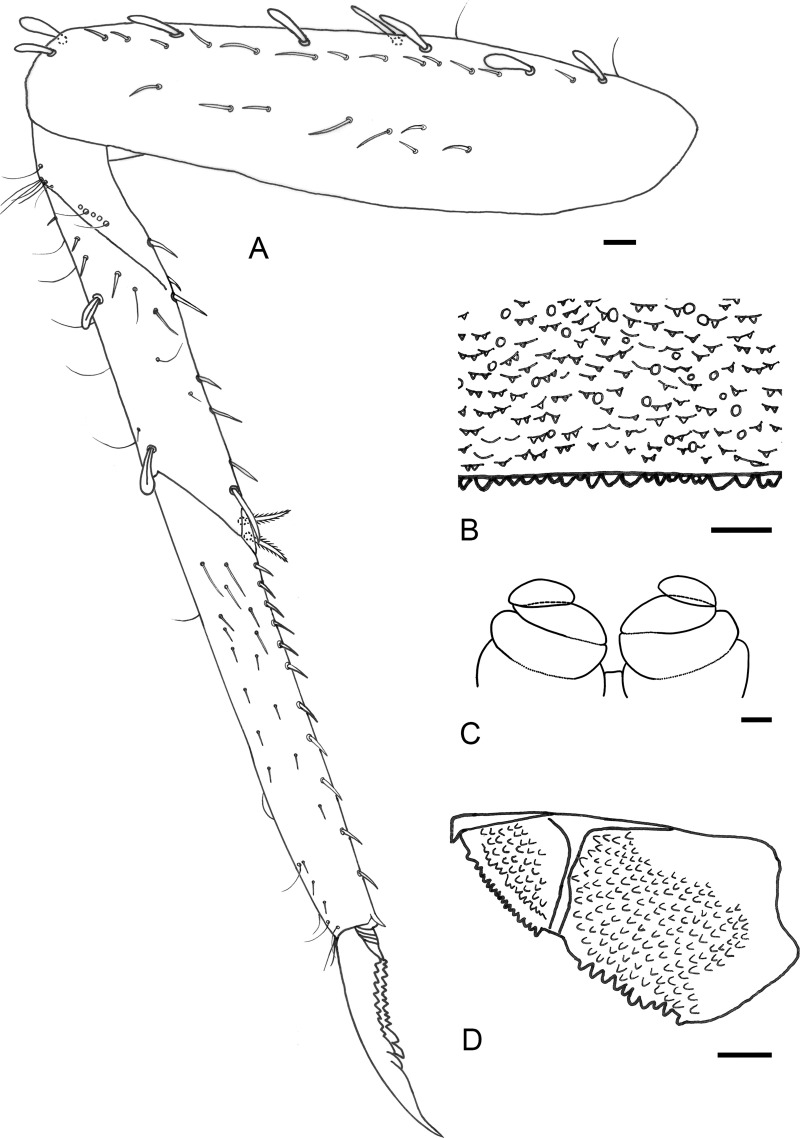
*Cymbalcloeon sartorii* gen. nov. *et* sp. nov. Larval morphology. (A**)** foreleg, (B) tergum VI, (C) gonostyli bud, (D) paraproct, Scale bar: 0.02 mm.

**Fig 6:**
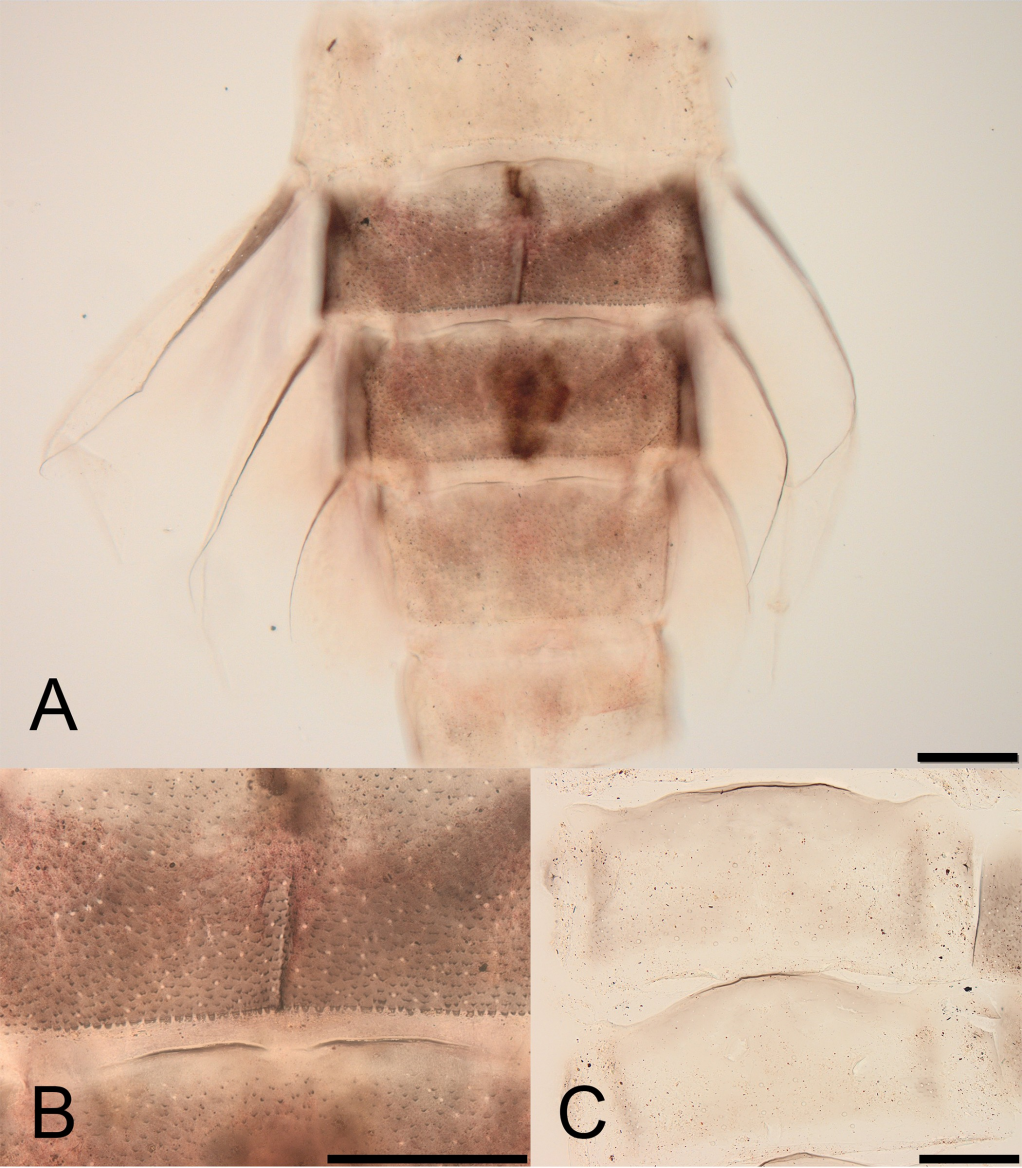
*Cymbalcloeon sartorii* gen. nov. *et* sp. nov. Larval morphology. (A) abdomen segments V–IX, (B) tergum VI, (C) sterna II–III. Scale bar: 0.1 mm.

Labrum (Fig 4A) rectangular, almost without distal emargination, dorsally covered with abundant setae not arranged in a row. Mandibles (Fig 4B and 4D) with margin between prostheca and mola deeply incurved, without setae; right prostheca bifid (Fig 4C). Maxilla (Fig 4G) with highly developed 2-segmented palp, long and curvate teeth at apex galea-lacinia. Labium (Fig 4H) with crescent shaped glossae and paraglossae covered with stout setae, labial palp (Fig 4I) with segments II and III almost fused, crescent shaped, covered with numerous very long, thin setae. Foreleg (Fig 5A), femur dorsally with few clavate setae, femoral patch absent; tibia with patella-tibial suture, tarsal claw elongated with two rows of denticles ending with two enlarged denticles. Hindwing pads absent. Three pairs of single gills on segments V–VI, ventrally oriented (Fig 6A). Gonostyli bud (Fig 5C) *Cloeon-*type.

#### Description

*Larva*.

### Head

*Antennae* long, ca. 4× as long as head width scape without process; scape and pedicel almost bare, without scales and scale bases; flagellum with triangular spines at apex of each segment. Base of antennae not close to each other and without carina.

*Labrum* (Fig 4A) rectangular, about twice larger than long; distal margin almost straight, with only a shallow median emargination; dorsal surface with long, simple setae, erratically distributed and not arranged in an arc; an arc of stout simple setae; ventral surface: distal margin with stout, simple setae.

*Right mandible* (Fig 4B and 4C). Canine with two almost completely fused sets of incisors, outer tooth of both sets much more developed than others, outer margin of outer set curved; prostheca slender, bifid with a very long inner branch, implantation of prostheca in depression on dorsal side; margin between prostheca and mola deeply concave without setae; mola area separated in two regions by a large tooth, apex of mola with set of thin setae.

*Left mandible* (Fig 4D and 4E). Canine with two almost completely fused sets of incisors, outer tooth of outer set much more developed than others, teeth of inner set equally developed, outer margin of outer set curved; prostheca robust, apically with small denticles and comb-shape structure, implantation of prostheca in depression on dorsal side; subtriangular process lateral to mola highly developed, base of outer margin with a bench of numerous setae, base of inner margin with small denticles; mola dorsally with well-developed denticles, apex of mola with set of thin setae.

*Hypopharynx* (Fig 4F). Lingua rounded, with almost straight apical margin covered with abundant minute setae; superlinguae rounded, distal margin with medium thin setae.

*Maxilla* (Fig 4G) slender, crown with three long, slender, curvate teeth and one denti-seta; three long setae at base of teeth; ventrally under crown, one row of scarce stout setae and one row with two pectinate denti-setae; 2-segmented maxillary palp, almost twice longer than galea-lacinia, segment I apically with a row of very long setae, segment II curved, apically pointed and with scarce very long setae.

*Labium* (Fig 4H and 4I). Glossae oblong, basally not expanded, apically rounded, longer than paraglossae, dorsal and ventral surfaces almost bare, single row of stout medium setae along outer and apical margins, single stronger seta at apex of glossae; paraglossae crescent shaped, dorsal surface and outer margin covered with long stout setae; labial palp 3-segmented, stout segment I as wide as long, segments II and III almost completely fused, dorsally and ventrally covered with numerous very long, thin setae, segment III conical, narrowing apically and ending with a short scale-like seta.

### Thorax

*Foreleg* (Fig 5A). Femur: dorsal margin with restricted number of long, clavate setae and a few long, thin setae, a row of medium setae parallel to dorsal margin, ventral margin almost bare, femoral patch absent; tibia: dorsal margin with long, thin setae, apex with single clavate seta, patella-tibial suture present, a row of long thin setae parallel to patella-tibial suture, ventral margin with a row of stout, medium to long setae ending with a group of stout long pectinate setae, laterally with numerous scattered spine-like setae; tarsus dorsal margin with a row of long thin setae, ventral margin with a row of stout, pointed medium setae, lateral surface with scattered spine-like setae; elongate, slender tarsal claw, slightly curved, with two rows of teeth increasing in length toward apex and ending broader teeth, subapical setae present (at least insertion visible).

Hindwing pads absent.

### Abdomen

*Terga* (Figs 5B, 6A and 6B). Distal margin of last terga with blunt spines.

*Sterna* (Fig 6C). Distal margin smooth, without spines.

**Fig 7:**
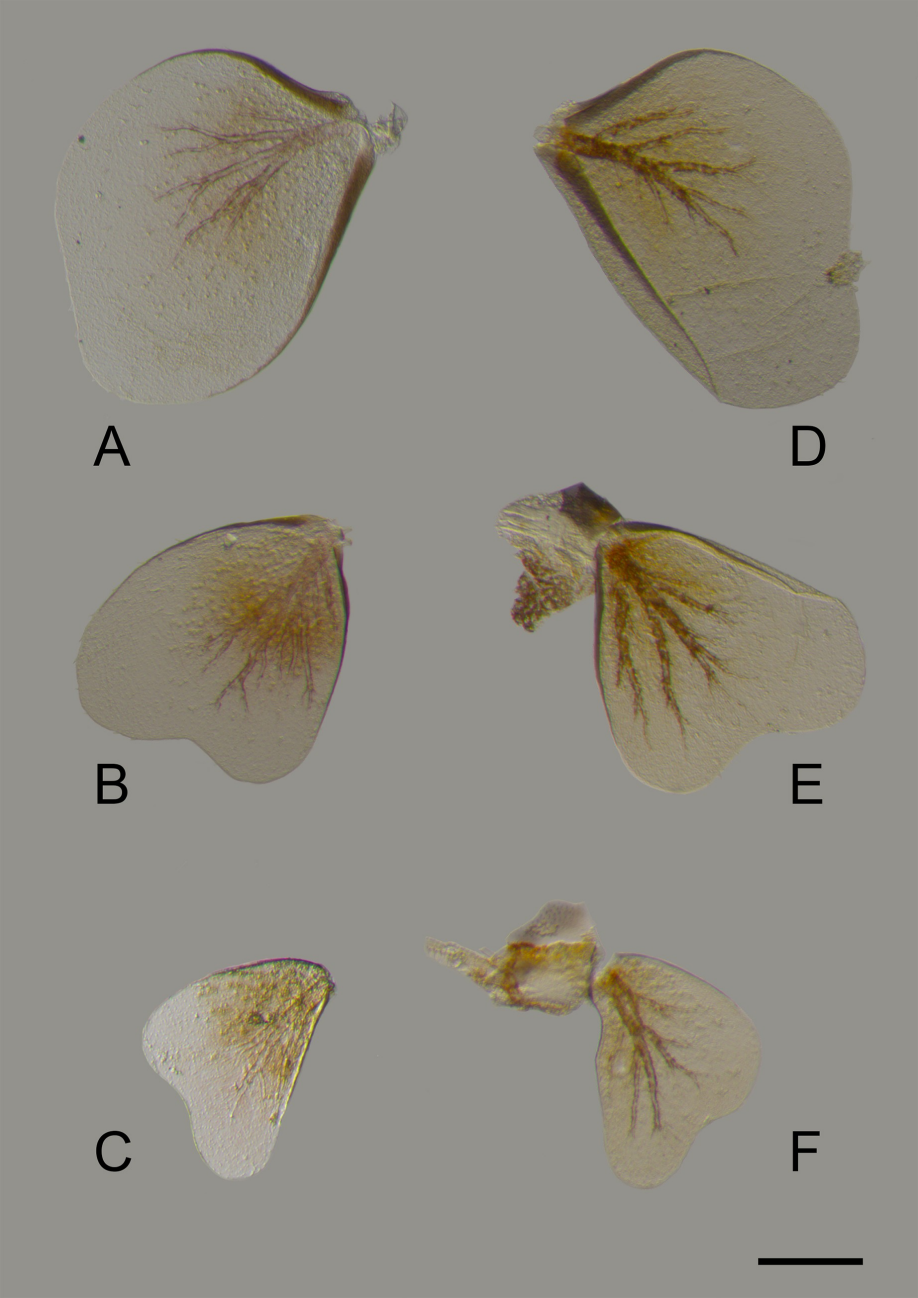
*Cymbalcloeon sartorii* gen. nov. *et* sp. nov. Gills morphology. (A) left gill V, (B) left gill VI, (C) left gill VII, (D) right gill V, (E) right gill VI (F) right gill VII. Scale bar: 0.1 mm.

*Gills* (Figs 6A and 7). Three pairs of single gills on segments V–VII, ventrally oriented covering sterna VI–IX, expanded, decreasing in size; Gill V asymmetrical, spoon shaped, tracheation well visible with ramification but restricted to central surface, subcostal and anal ribs present; gill VI heart-like shape, costal and anal ribs present, tracheation well developed; gill VII similar to gill VI except only costal rib present.

*Gonostyli bud* (Fig 5C). Three segments. Segment II cylinder directed laterally; segment III curved, directed medially.

*Paraproct* (Fig 5D). Distal margin with poorly developed triangular spines, without prolongation at posterior margin, surface shagreen; cercotractor with triangular marginal spines, surface shagreen.

*Caudal filaments*. Inner margin of cerci with very thin, long setae; median caudal filament equal to cerci, bilateral, with very thin, long setae.

#### Imago

Unknown.

#### Biological aspects

The near-synchronous gill movements observed in resting stage are similar to the beating of a cymbal instrument (S1 Video). The average rate of gill movements is 11.69 times/sec. (range 8.77–14.50, n = 5).

#### Distribution

Thailand.

#### Etymology

The name of this genus is an arbitrary combination of the “*Cymbal*”–a musical instrument similar in appearance to the gill plate and gill mobility, and “*Cloeon*”–the most common and widespread genus of the subfamily. The gender is neutral.

### *Cymbalcloeon sartorii* sp. nov.

urn:lsid:zoobank.org:act:E21778A6-FDE0-4303-B3D1-F5889E1E9F99

#### Material examined

**HOLOTYPE**: male larva in ethanol, THAILAND, Kanchanaburi province, Thong Pha Phum district, Huai Khayeng stream, Ban Pra Chum Mai, 14°34’57.9”N, 98°34’52.0”E, 269 m, 20.XII.2019, B. Boonsoong leg. (ZMKU). **PARATYPES**: 3 larvae on slide and 1 larvae in ethanol (ZMKU), 1 larva in ethanol (MZL); voucher: GBIFCH00673236, same data as holotype; 1 larva in ethanol, same data as holotype, 12.VII.2020 (ZMKU); 3 larvae on slide and 2 larvae in ethanol (ZMKU), 1 larva on slide (MZL); voucher: GBIFCH00673096, THAILAND, Loei province, Phu Luang district, Ban Non Pattana, 17°06’24.0”N, 101°28’43.4”E, 527 m, 18.XII.2018, C. Suttinun leg.

#### Description

*Larva* (Figs 1 and 2). Body length 2.5–2.9 mm.

*Coloration* (Figs 2 and 3). Head dorsally light yellow with medium brown vermiform marks, head and thorax with bright, median, dorsal suture. Thorax dorsally dark brown with a few diffusive light orange maculae on pleura medially and a few diffusive blackish close to median suture. Abdomen: terga I to III medium brown with a symmetrical large yellow spot distally, terga IV and V yellow, terga VI and VII dark brown, terga VIII to X yellow with light brown pattern. Head ventrally yellowish, thorax ventrally light brown, abdomen ventrally yellowish with sterna VI and VII darker. Legs whitish. Caudal filaments yellowish with darker band.

### Head

*Antenna* (Fig 3A). ca. 21 segments; flagellum with triangular spines at apex of each segment.

*Labrum* (Fig 4A). Rectangular, length 0.6× maximum width. Dorsal surface with long, fine, simple setae erratically distributed and not arranged in an arc; an arc of five stout, simple setae anteromedially along distal margin. Ventral surface medially with medium stout setae; an arc of ten stout, simple setae laterally and anterolaterally.

*Right mandible* (Fig 4B and 4C). Canine with 4 + 3 denticles; prostheca with short branch apically denticulated, long branch with one denticulated margin and one bare margin; mola with reduced denticulation.

*Left mandible* (Fig 4D and 4E). Canine with 3 + 3 denticles; prostheca apically with small denticles and a comb-shape structure; mola with well-developed denticulation.

*Hypopharynx* (Fig 4F). Lingua equal to superlingua, rounded with apical almost straight margin covered with abundant short setae.

*Maxilla* (Fig 4G). Galea-lacinia without setae under crown. Medially without setae. Maxillary palp 2× as long as length of galea-lacinia; palp segment II 1.9× length of segment I; setae on maxillary palp very long, fine, simple, scattered over surface of segments I; segment II curved, apically pointed and with scarce very long setae.

*Labium* (Fig 4H and 4I). Glossae: dorsal and ventral surfaces almost bare, with 15 medium, stout setae along outer and apical margin. Paraglossae dorsal surface covered with 6–7 long, stout setae not arrange in row and outer margin covered with single row of 9–10 long, stout setae; apex with a long, stout seta; ventrally bare. Labial palp with segment I 0.5× length of segments II and III combined.

### Thorax

#### *Hindwing pads* absent

*Foreleg* (Fig 5A). Ratio of foreleg segments 1.6:1.0:1.0:0.3. Femur. Length 4× maximum width; dorsal margin with a row of ca. six long, clavate setae with long, thin setae, apex with two long, curved, clavate setae; length of setae 0.4× maximum width of femur. Tibia. Dorsal margin with long a row of ca. six thin setae; patella-tibial suture present, two groups of ca. four long, thin setae parallel to patella-tibial suture; ventral margin with a row of ca. six stout, medium to long setae ending with two stout, long, pectinate setae; Tarsus. Dorsal margin with a row of ca. seven long, thin setae; ventral margin with a row of 11–13 stout, pointed, simple setae. Tarsal claw with two rows of many minute denticles and two broad quadrangular teeth increasing in length toward apex.

*Middle leg* and *hind leg*. As foreleg.

### Abdomen

*Terga* (Figs 5B and 6B). shagreen, few very long thin setae scattered over surface. Posterior margin of segment I smooth, without spines, segments II–IX with blunt spines.

*Sterna* (Fig 6C). Distal margin without spines or structure; surface laterally shagreen.

*Gills* (Figs 6A and 7). Gill V as long as length of segments VI to ½ IX; gill VI as long as length of segments VI to IX; gill VII as long as length of segments VII to IX.

*Gonostyli bud* (Fig 5C). Three segments. Segment III 0.7x length of segment II.

*Paraproct* (Fig 5D). Posterior margin with ca. 10 poorly developed triangular marginal spines, without prolongation at posterior margin; cercotractor with numerous triangular marginal spines.

*Caudal filaments* (Fig 4). Cerci ca. 0.4× body length, median caudal filament equal to cerci.

#### Etymology

This species is dedicated to Dr. Michel Sartori (Museum of Zoology, Lausanne) for his outstanding contributions to the taxonomy of mayfly fauna worldwide and more recently in Thailand.

#### Distribution

Thailand.

#### Biological aspects

**Fig 8:**
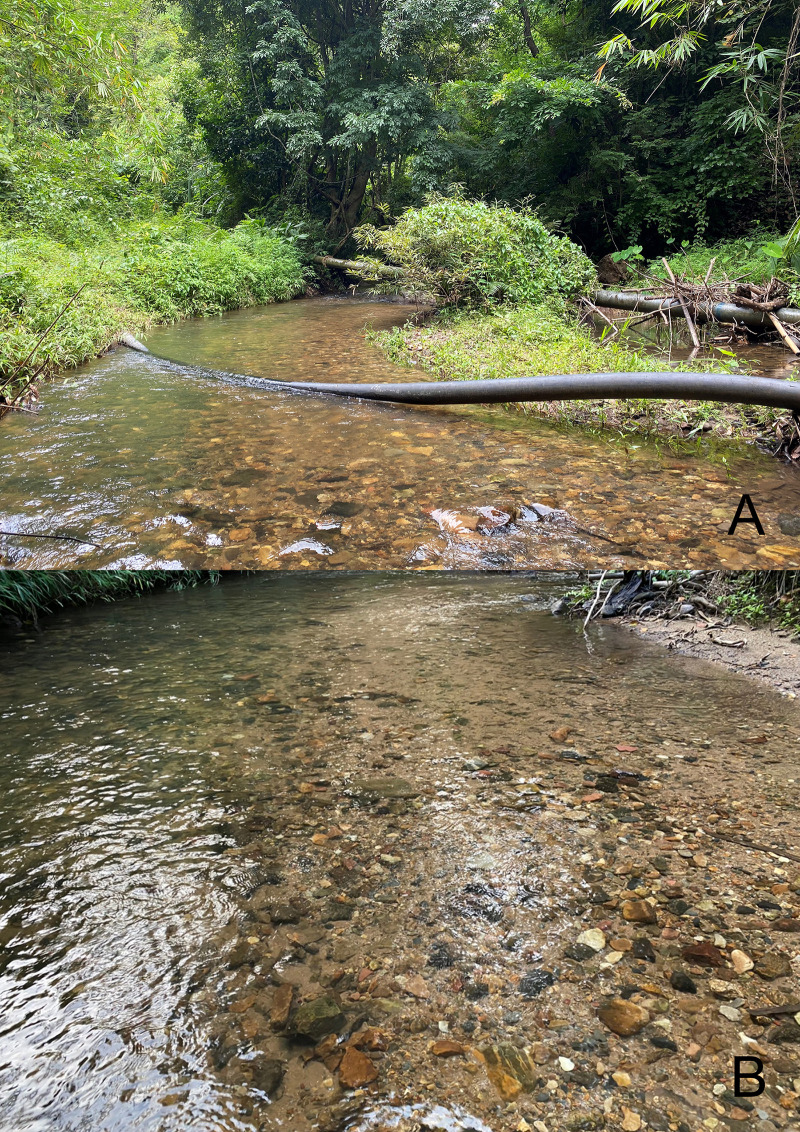
*Cymbalcloeon sartorii* gen. nov. *et* sp. nov. Habitat. (A) sampling site, Huai Khayeng stream, Kanchanaburi province, Thailand, (B) microhabitat.

The specimens were collected at altitudes from 265 m a.s.l. to 530 m a.s.l. in two small, shallow, slow headwater streams (Fig 8). Larvae were found in sandy and pebble bottom substrates, usually together with larvae of *Nigrobaetis* Novikova & Kluge, 1987 and *Centroptella* Braasch & Soldán, 1980.

## Discussion

Despite presenting unusual and very derived characters, *Cymbalcloeon* gen. nov. clearly belongs to the Baetidae, based on its fish-like body shape, relatively long antennae originating anterolaterally on the head, Y-shaped epicranial suture reaching ventrally of lateral ocelli, labium with long and narrow glossae and paraglossae, and the developing turbinate eyes of the male larvae [8]. This genus can be assigned to Anteropatellata because of the presence of a patella-tibial suture on the foreleg and to non-Baetovectata because the gonostyli bud of the male ready-to-molt larva has the 2^nd^ segment of the gonostyli bud directed laterally and a medially curved 3^rd^ segment (“*Cloeon*-type” not the “*Nigrobaetis*-type”) [9, 10]. Claws possess two rows of small denticles ending with two larger ones, which clearly excluded the belonging of this genera to Baetungulata.

As the larva does not present spines on the lateral margin of any abdominal segments and both mandibles do not have setae between prostheca and mola, the genus differ from *Cloeon* and all the related genera (*Cloeon* complex sensu Hill & Pfeiffer & Jacobus 2010 or Cloeon/fg1 sensu Kluge [11]). This new genus could be closely related to the *Baetopus/*g1 sensu Kluge [12] based on the following characters: 1) labial palp with segment II and III thickened, almost completely fused, segment III nipple-like; 2) maxillary palp is two-segmented, segment II much more developed; 3) mandibles without setae between prostheca and mola; 4) lateral sides of larval abdomen without spines; 5) cerci without dark rings on each 4^th^ joining segment [13]. However, *Cymbalcloeon* gen. nov. can be easily distinguished from *Baetopus*/g1 by the unique gills. These three pairs of single gills are present on segments V–VII, ventrally oriented to cover sterna VI–IX, expanded, decreasing in size, and the mobility of gills. The shape of the gills is an autapomorphy, unique among other Baetidae.

Baetopus/g1 is divided into *Baetopus* Keffermuller, 1960 and *Raptobaetopus* Müller-Liebenau, 1978 [12]. Based on the mouth apparatus, *Cymbalcloeon* gen. nov. seems more closely related to *Baetopus* Keffermuller, 1960 than *Raptobaetopus* Müller-Liebenau, 1978. In *Cymbalcloeon* gen. nov. and *Baetopus* Keffermuller, 1960, mouth apparatus is less specialized; labrum with incision; glossa is not narrowed in proximal part. Mouth apparatus of *Raptobaetopus* Müller-Liebenau, 1978 is modified and deeply adapted to carnivorous behavior; labrum without median incision; mandibles with long and pointed canine and reduction of mola area; glossae are narrowed in proximal part. There are a few characters of *Cymbalcloeon* gen. nov. similar to *Raptobaetopus* Müller-Liebenau, 1978 including: 1) the shape of labial palp segments II and III which are thicker near base, narrowing toward apex; 2) long, pointed teeth of maxillary being pressed together, forming a claw-like structure [14–16]. Unlike *Baetopus* and *Raptobaetopus* which have claws with two very short rows of minute denticles, *Cymbalcloeon* possesses claws with two well developed rows of denticles. *Cymbalcloeon* presents also unexpected similarities with the Neotropical genus *Adebrotus* Lugo-Ortiz & McCafferty, 1995; especially the mola of both mandibles, the right prostheca, the shape and setation of legs and claw of *Adebrotus amazonicus* Lugo-Ortiz & McCafferty, 1995 (Figs 3 and 4 in [17]; Fig 13 in [18]), the shape of the labium palp, shape and setation of the labrum of *Adebrotus lugoi* Salles, 2010 (Figs 17 and 24 in [18]). The two genera differ by the presence of setae between prostheca and mola of the left mandible, two deeply cleft sets of incisors on right and left mandibles in *Adebrotus*. The genus *Adebrotus* also presents unmodified gills on segments I to VII. It remains difficult to decide if the noticed similarities are due to strict convergences or reflect true relationships between the two genera. The position of *Adebrotus* within the Baetidae also remains unclear. Kluge [19], in his non-ranking phylogeny of mayflies, did not include the genus. In a cladistical analysis of South American Baetidae based on morphological characters only [20], *Adrebotus* was recovered in a rather basal position, sister group, but without support, of a clade including *Paracloeodes* Day, 1955, *Waltzohyphus* Lugo-Ortiz & McCafferty, 1995 and *Callibaetis* Eaton, 1881.

Legs of *Cymbalcloeon* also present similarities with those of *Indocloeon* Müller-Liebenau, 1982: elongated claw with two rows of denticles ending with two greatly enlarge ones, as well as the presence of bipectinate setae on the ventral margin of tibiae. *Indocloeon* belongs to Protopatella as the patella-tibial suture is present on middle and hind legs only [9]. Other characters, especially on mouthparts, also clearly indicate that the two genera are most probably not related and the similarities in legs should be due to convergences.

The knowledge of the imaginal stages will be of great importance for the position of *Cymbalcloeon* within the Baetidae. According to our previous statements, the forewings have most certainly single intercalary veins, but it must verified by rearing or association of life stages by barcoding. The shape and structure of gonopods, gonovects and other male genital structures will be also of great phylogenetic importance. As shown in most recent reconstructions [20, 21], the imaginal characters are scarcer than larval ones in Baetidae, but are more conservative and less subject to convergences.

The most astonished character of *Cymbalcloeon* is obviously its strange gills: they are present on segment V–VII only and absent on segments I–IV. Moreover, the shape and movement are unique within the family. Noticeably, the gill movements in the family Baetidae are limited, at our knowledge, to the *Cloeon* complex and to species previously attributed *Afroptilum* [22]. According to Gillies [22], gill movement should be considered as an adaptation by mayflies to the water with lower current speeds. Our preliminary observations in Thailand indicated no gill movements in *Labiobaetis* and *Nigrobaetis*, but gill movements were observed in *Cloeon* Leach, 1815, *Centroptilum* Braasch & Soldán, 1980, and *Procloeon* Bengtsson, 1915. Gills of *Cloeon* sp. (lentic species) move twenty times more slowly (average 0.56 times/sec.; range = 0.31–0.83, n = 3) than those of the new genus. The new genus can be found in slow-to-moderate currents with sand and pebble bottoms, where we also collected *Nigrobaetis* and *Centroptella* which observed in field without gill moving abilities. The reduction of gills of *Cymbalcloeon* might be the reason why the gills is moving and moving faster than *Cloeon* sp. (lentic species). However, the behavior of the new genus is identical to that of the genus *Cloeon*, which has a streamlined cylindrical body.

## Supporting information

S1 Video *Cymbalcloeon sartorii* gen. nov. *et* sp. nov. Gills movements. (MOV)

## Abbreviations

ZMKU: Zoological Museum of Kasetsart University, Bangkok (Thailand)
MZL: Museum of Zoology, Lausanne (Switzerland)
